# An Efficient and Economical *N*-Glycome Sample Preparation Using Acetone Precipitation

**DOI:** 10.3390/metabo12121285

**Published:** 2022-12-17

**Authors:** Junyao Wang, Wenjing Peng, Mojibola Fowowe, Oluwatosin Daramola, Yehia Mechref

**Affiliations:** Department of Chemistry and Biochemistry, Texas Tech University, Lubbock, TX 79409, USA

**Keywords:** glycan, permethylation, acetone precipitation, oxidative release, LC-MS/MS

## Abstract

Due to the critical role of the glycome in organisms and its close connections with various diseases, much time and effort have been dedicated to glycomics-related studies in the past decade. To achieve accurate and reliable identification and quantification of glycans extracted from biological samples, several analysis methods have been well-developed. One commonly used methodology for the sample preparation of *N*-glycomics usually involves enzymatic cleavage by PNGase F, followed by sample purification using C18 cartridges to remove proteins. PNGase F and C18 cartridges are very efficient both for cleaving *N*-glycans and for protein removal. However, this method is most suitable for a limited quantity of samples. In this study, we developed a sample preparation method focusing on *N*-glycome extraction and purification from large-scale biological samples using acetone precipitation. The *N*-glycan yield was first tested on standard glycoprotein samples, bovine fetuin and complex biological samples, and human serum. Compared to C18 cartridges, most of the sialylated *N*-glycans from human serum were detected with higher abundance after acetone precipitation. However, C18 showed a slightly higher efficiency for protein removal. Using the unfiltered human serum as the baseline, around 97.7% of the proteins were removed by acetone precipitation, while more than 99.9% of the proteins were removed by C18 cartridges. Lastly, the acetone precipitation was applied to *N*-glycome extraction from egg yolks to demonstrate large-scale glycomics sample preparation.

## 1. Introduction

Glycosylation has been recognized as one of the major processes that occurs during the post-translational modifications (PTMs) of proteins [1,2]. More than half of the proteins found in the human body have been confirmed as glycosylated [3,4,5,6]. By adding glycan structures onto the peptide backbone, the properties and functions of a protein can be greatly altered [7,8]. Glycans are also known to impact various biological functions, such as immune response [9] and intercellular signaling and interactions [10,11,12]. The different functions are mainly related to the high diversity of glycan structures [6,13,14], which involve various types of isoforms [15,16,17]. In addition, glycan expression changes have been reported in numerous diseases including cancer (breast cancer [18], colorectal cancer [19], liver cancer [20]), immunity deficiency [21], and hereditary disorders [9], making the corresponding glycans potential disease markers for diagnostics. Moreover, glycans are essential components of many small-molecule drugs such as antibiotics and anticancer therapeutic agents. Patients with metabolic diseases, such as congenital glycosylation disorders, can benefit greatly from oral supplementation of monosaccharides, indicating the therapeutic efficacy of glycans [22].

The analytical strategy for glycome profiling usually includes the extraction of glycans followed by instrumental analyses [23,24]. Enzymatic cleavage has been commonly used by researchers to remove glycans from glycoproteins. Specifically, peptide *N*-glycosidase F (PNGase F) is a widely used enzyme which can release most *N*-glycans with free reducing ends [25]. However, enzymatic release is practical with only a small quantity of samples, on a scale of micrograms or microliters. Adding elevated units of PNGase F to a large volume of samples not only highly increases the cost of an experiment, but also potentially decreases the releasing efficiency of the enzyme. In addition, PNGase F digestion usually takes a long time, ranging from hours to overnight. Cummings and coworkers introduced an oxidative strategy to release glycans from different types of biological samples using household bleach [26], with an effective ingredient of sodium hypochlorite (NaClO). It is known that NaClO can degrade proteins [27,28,29], and also effectively degrade glycoproteins to release free glycans, including the core-fucosylated *N*-glycans with α1,3 linkage which are resistant to PNGase F [30]. The labile groups, e.g., sialic acid can also be preserved [26]. Moreover, using NaClO as a substitute for PNGase F can greatly reduce experimental time and costs, while simultaneously increasing the capacity of sample preparation.

Mass spectrometry is considered a powerful tool for glycomics analysis due to its high sensitivity, and MS^n^ scans can provide fragmental information of glycans to boost structural identification [31]. However, the MS signal is mainly driven by the ionization efficiency of the analytes [32], making it very necessary to perform purification or enrichment processes prior to MS analysis. By removing the non-targeted species such as proteins or peptides, the competitive ionization can be greatly suppressed, thereby enhancing the signals of glycans [33,34,35]. Regarding the purification methods for protein removal, according to the varied hydrophobicity between proteins and glycans, C18 cartridges have become a popular option to separate the hydrophobic proteins from the samples after glycan release [36,37]. Recently, Guan et al. compared purification efficiency of C18 cartridges with size exclusive (10 kDa) filtration and 90% ethanol precipitation [38]. According to their results, 90% ethanol had low efficiency to remove de-*N*-glycosylated proteins, and size exclusive filtration yielded least amount of abundant *N*-glycans. On the other hand, C18 cartridge cleanup provided a higher signal-noise ratio than the other two methods, making it an optimal approach to prepare high purity glycan samples. However, similar to PNGase F, the sample processing quantity for each C18 cartridge can be limited by the volume capacity of the spin column. Recently, acetone precipitation was demonstrated to purify glycans from standard glycoproteins [39]. Additionally, it has been employed for the enrichment of glycopeptides [40].

In this study, acetone was used to precipitate *N*-glycans extracted from bovine fetuin and complex human serum samples. The performance was assessed at lab scale by comparing it with C18 cartridge cleanup. Acetone precipitation is not limited by the volume capacity of C18 cartridges. Moreover, using solid phase extraction (SPE) cartridges can lead to potential leachables due to the direct contact between packing materials and the sample. Our results indicate that acetone precipitation is more efficient to yield significantly higher intensity of multiple complex *N*-glycans. However, the major downside is for smaller glycans since they are not sufficiently hydrophilic to fully move into the aqueous layer. In addition, the performance of protein removal was slightly lower. Additionally, acetone precipitation was combined with oxidative release to aid the bulk production of glycans from egg yolks. This is a less costly method which requires neither enzymes nor SPE cartridges. Therefore, it may become an economical approach to produce a large amount of glycan crudes from natural products.

## 2. Results and Discussion

The purification of *N*-glycans using acetone precipitation was validated in two aspects. Firstly, after being treated by acetone, the *N*-glycan abundance from 1 μg bovine fetuin and 1 μL human serum was compared against the same amount of samples filtered by C18 cartridges to study if acetone precipitation would achieve a high glycan yield. Secondly, the proteomics analyses were performed to assess the efficiency of removing proteins from the samples.

### 2.1. Glycomics Analysis

**Figure 1:**
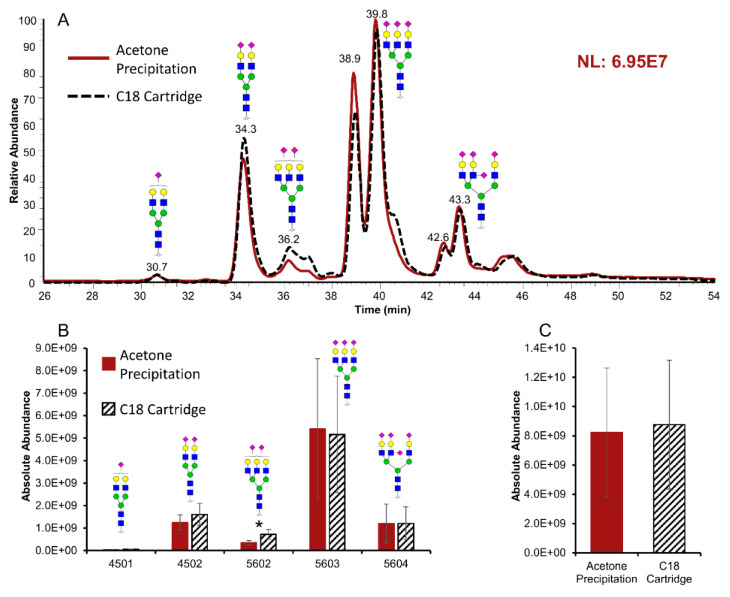
(**A**) Extracted Ion Chromatograms (EICs) of permethylated *N*-glycans derived from bovine fetuin treated by acetone precipitation (red trace) and C18 cartridges (black trace). (**B**) Quantification of *N*-glycans based on the area under the peak (n = 3, *: *p* < 0.05). (**C**) Total abundance of *N*-glycans detected in the samples prepared by acetone precipitation and C18 cartridges. Symbols: 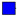, *N*-acetylglucosamine (GlcNAc); 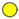, Galactose (Gal); 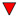, Fucose (Fuc); 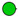, Mannose (Man); 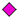, *N*-acetylneuraminic acid (NeuAc/Sialic Acid).

As a standard glycoprotein, the peptide sequence and glycan compositions of bovine fetuin have been fully characterized [41,42]. Figure 1A provides the EICs of detected *N*-glycans derived from 1 μg of bovine fetuin. To highlight the intensity differences, the scale of the *y*-axis was fixed to the higher intensity of the two traces. The red trace represents the sample treated by acetone, while the black trace represents the sample treated by the C18 cartridge. After analyzing three replicates of both treatments, the identified *N*-glycans were quantified by calculating the area under their corresponding peaks using Xcalibur 4.4 software. The average values of the triplicates were used to generate the bar graph in Figure 1B, where the error bars denote the standard deviations. According to the Student’s t-test, the structure of HexNAc_5_Hex_6_NeuAc_2_ (5602) was found with significantly higher abundance after the C18 cartridge filtration compared to acetone precipitation (*p* < 0.05). No statistical significance was observed in the other four detected structures. The total abundance of all detected *N*-glycans was summed and compared in Figure 1C. Although the average of absolute abundance after acetone precipitation (8.23 × 10^9^) is slightly lower than C18 cartridges (8.76 × 10^9^), the difference is not significant (*p* > 0.05).

To further investigate the applicability of acetone precipitation on complex biological samples, *N*-glycomics samples derived from human serum were prepared. Different types of *N*-glycans such as high mannose, fucosylated, and sialylated structures are normally found in human serum, making it a good choice to examine whether acetone tends to selectively enrich certain types of *N*-glycans. However, since human serum contains many unknown glycan structures, the raw data files were first processed using Multiglycan 1.5 software [43], with the mass tolerance set at 10 ppm. The software generated a list of identified glycans based on the given raw data files. Then, each software-identified glycan was manually checked against their EICs and MS results using Xcalibur 4.4. The manual checking process is demonstrated by Figure S2. The first example is an *N*-glycan with a composition of HexNAc_4_Hex_5_Fuc_1_ (4510). The theoretical *m*/*z* of protonated 4510 after reduction and permethylation is 1119.5905. By entering this *m*/*z* into Xcalibur, the EIC of the structure is acquired with the peak at 27.6 min. The full MS spectrum given by the inset of Figure S2A presents the monoisotopic peak with m/z of 1119.5863, which indicates the mass accuracy of 3.75 ppm. Figure S2B shows the MS/MS spectrum of 4510. The fragment ion with m/z of 468.1 is considered a diagnostic ion to confirm that the fucose is connected to the core of this *N*-glcyan. Following the same strategy, the EIC and MS spectra of sialylated structure HexNAc_4_Hex_5_NeuAc_2_ (4502) are provided in Figure S2C,D.

**Figure 2:**
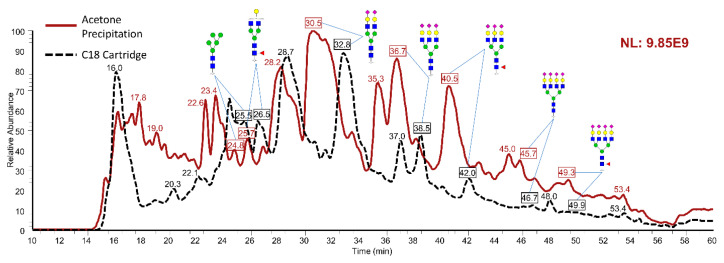
TICs of *N*-glycomics samples derived from 1 μL of human serum after purified by using acetone (red trace) and C18 cartridges (black trace). Symbols of monosaccharides are the same as shown in Figure 1.

**Figure 3:**
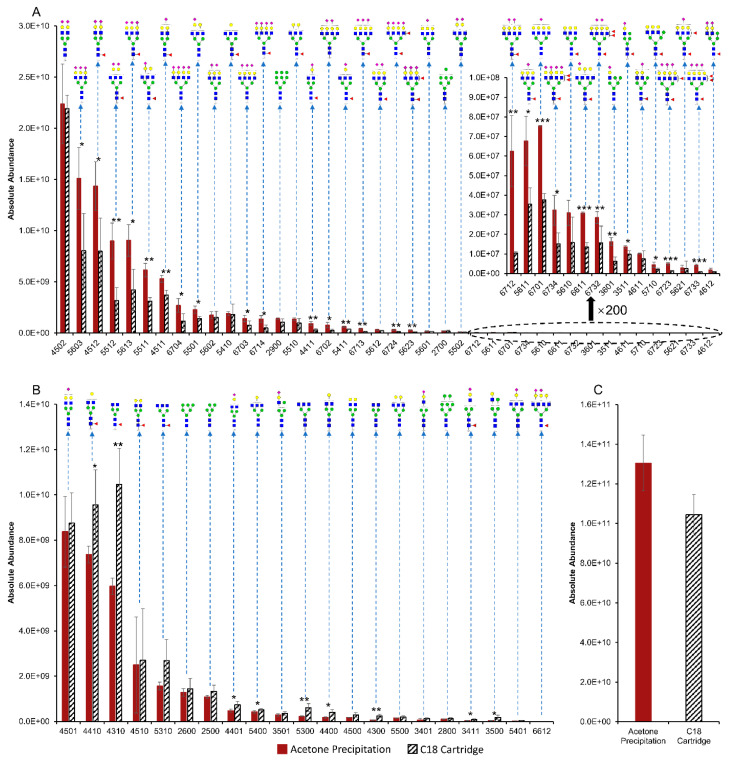
Comparisons of 61 *N*-glycan abundances from 1 μL of human serum treated by acetone precipitation and C18 cartridges. (n = 3) (**A**) The *N*-glycans showing higher average abundances purified by acetone. (**B**) The N-glycans showing higher abundances purified by C18 cartridges. (**C**) Abundances of total *N*-glycans. *: *p* < 0.05, **: *p* < 0.01, ***: *p* < 0.001. Symbols of monosaccharides are the same as shown in Figure 1.

Overall, a total of 61 *N*-glycans derived from human serum were identified and quantified. Figure 2 provides the TICs of permethylated *N*-glycans extracted from 1 μL of human serum, with identified *N*-glcyans labeled next to the corresponding peaks. Again, the *y*-axis uses a fixed scale to compare the peak intensities between acetone- and C18-treated samples. The TIC of acetone precipitation (red trace) exhibited relatively higher abundance than C18 cartridges (black trace) on several *N*-glycans. Similar to bovine fetuin, three preparation replicates using either method were analyzed. Then, the average abundance of each structure was compared between the two methods. Among the 61 *N*-glycans, 40 structures were observed with higher abundance after acetone precipitation than after treatment with C18 cartridges (Figure 3A). In addition, Student’s t-tests suggested that 27 out of the 40 structures had significantly higher abundance (*p* < 0.05), and those are labeled with asterisks. On the other hand, 21 *N*-glycans were found with lower abundance after acetone precipitation as shown in Figure 3B, where nine structures are labeled as statistically significant. Focusing on the significant *N*-glycans from human serum, we observed that all the structures with higher abundance in acetone precipitation were sialylated, such as HexNAc_5_Hex_6_NeuAc_3_ (5603), HexNAc_4_Hex_5_Fuc_1_NeuAc_2_ (4512), and HexNAc_6_Hex_7_NeuAc_4_ (6704). One major explanation for these results could be related to the higher hydrophilicity of sialic acid relative to other monosaccharides, making it easier to be precipitated by organic solvent. On the other hand, the *N*-glycans that showed significant down-regulations of their abundances in acetone precipitation were mostly neutral structures, such as HexNAc_4_Hex_4_Fuc_1_ (4410) and HexNAc_5_Hex_3_Fuc_1_ (5310). These results are also comparable with the previously reported study by Valk-Weeber et al. [39], suggesting that acetone precipitation is more suitable for complex and sialylated *N*-glycans. However, for smaller neutral *N*-glycans, acetone precipitation is not the best option for purification since it can cause more sample loss.

The total serum *N*-glycan abundance was also calculated and is presented by Figure 3C. Because almost two thirds of the structures exhibited higher abundance in acetone precipitation, the total abundance was also observed with significant up-regulation (*p* = 0.03). Therefore, acetone precipitation may be a better option than C18 cartridges in terms of *N*-glycan yield from human serum, especially when it comes to the purification of sialylated *N*-glycans.

### 2.2. Proteomics Analysis

Apart from glycomics analyses, we also compared the purification performance of acetone and C18 from another approach by analyzing the protein abundance in the samples after using the two methods. First, the standard glycoprotein bovine fetuin was again used as an example. Figure S3A provides the TIC of 1 µg of tryptic digested bovine fetuin without protein removal treatment. Figure S3B,C present the TICs of the 1 µg of tryptic digested bovine fetuin after acetone precipitation and C18 cartridge treatment, respectively. With the scale of the *y*-axis fixed to the highest intensity of 9.32 × 10^9^, the second and third TICs appear as flat lines because the intensity of the two chromatograms are lower than 1% of 9.32E9, indicating that both methods can effectively remove more than 99% of the peptides from tryptic digested bovine fetuin. To further compare the performance between acetone and C18, Figure S4A,B highlight the TIC differences. The quantified comparison was also performed by processing the data files using Scaffold 3.6.3 software. With the Mascot score set at 25 and protein identification confidence at 99%, the software reported quantification results in term of spectra counts for each file. Figure S4C shows 34 and 42 spectra counts of 1 µg bovine fetuin treated by acetone precipitation and C18 cartridges, respectively, with no significant difference between the two methods. According to our quantification result of *N*-glycans and protein spectra counts of bovine fetuin, acetone precipitation provided similar sample purification performance to the C18 cartridges for this specific type of protein.

To gain a more comprehensive understanding of protein removal efficiency using acetone, human serum was selected as an example of a complex biological sample, as it contains hundreds of different types of proteins [44]. Based on the protein concentration from the BCA protein assay, the human serum sample containing 1 µg of tryptic digested proteins was subjected to LC-MS analysis and generated the TICs in Figure S5A. The protein quantification was performed using MaxQuant 2.0.3 with the parameters set as follows: miscleavage allowance of trypsin was set to 2; the shortest peptide for protein identification was 6; the mass tolerance for peptide precursor and fragment ions were set at 20 ppm and 0.5 Da, respectively; the false discovery rate (FDR) was 0.01; and protein abundance was quantified based on the peak area. As shown in Figure S5B, compared to the non-treated sample, the total protein abundance was remarkably reduced after using the two purification methods. On average, acetone was able to remove 97.7% of the proteins, whereas the C18 cartridge showed a more thorough clean-up with more than 99.9% of proteins removed. This result can also be observed by the TICs in Figure S6, where multiple peaks were detected, especially between the retention time window of 70 to 100 min, in the samples prepared by acetone precipitation. However, the other samples prepared by C18 cartridges showed a few peaks between 18 and 50 min. In RPLC, the retention time is mainly driven by the hydrophobicity of the analytes [45], and a longer retention time usually corresponds with higher hydrophobicity. These chromatograms suggest that during the purification processes, C18 has stronger affinity with the more hydrophobic peptides than acetone, thereby leading to the very low intensity on the right side of the TIC in Figure S6B.

### 2.3. N-Glycomics of Egg Yolks

**Figure 4:**
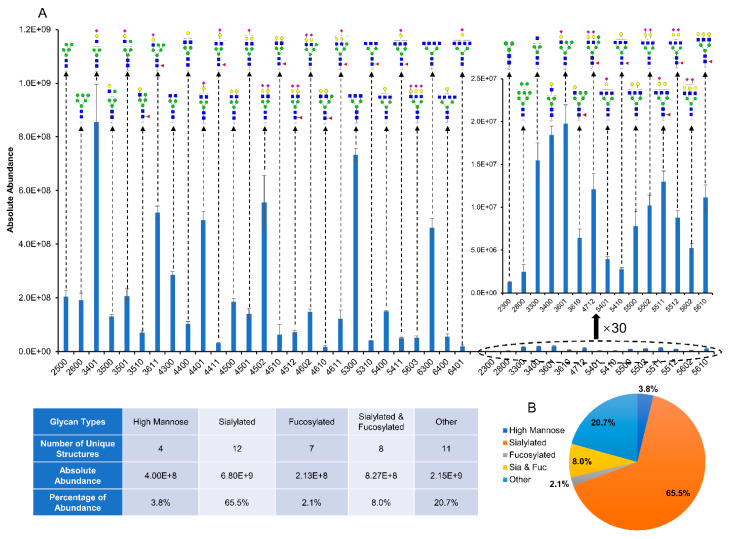
Quantification results of *N*-glycans extracted from egg yolks using oxidative release followed by acetone precipitation. (**A**) Absolute abundance of 42 identified *N*-glycans. (**B**) Distribution of five types of *N*-glycans. Symbols of monosaccharides are the same as shown in Figure 1.

After validating the glycan purification performance on standard glycoprotein bovine fetuin and complex human serum samples, acetone precipitation was applied for the enrichment of *N*-glycans derived from egg yolks. The purpose of this experiment was to demonstrate the potential application of acetone precipitation for a large-scale production of glycans from natural products without enzymatic cleavage. The rationale for combining oxidative release with acetone precipitation is that after glycan release, there are *N*-glycans, amino acids, and short peptides in the mixture. Previous methods used C18 and size-exclusive columns to purify *N*-glycans, which are costly. Since amino acids can be easily removed by dialysis, and *N*-glycans are more hydrophilic than peptides, acetone precipitation can be an alternative to purify *N*-glycans from peptides. After *N*-glycan release using 8.25% NaClO solution, followed by dialysis and acetone precipitation for sample purification, a total of 1.44 g crude glycans were collected from three egg yolks. Then, an aliquot of resuspended sample containing 40 µg of crude glycans was injected for LC-MS/MS analysis. Using the same identification and quantification methods as already described, 42 unique *N*-glycans were identified as shown in Figure 4A. The structures with high abundances were either sialylated, such as HexNAc_3_Hex_4_NeuAc_1_ (3401) and HexNAc_4_Hex_5_NeuAc_2_ (4502), or neutral structures without sialic acid and fucose, such as HexNAc_5_Hex_3_ (5300) and HexNAc_6_Hex_3_ (6300). The full list of these 42 *N*-glycans is provided by Table S1, with mass accuracy less than 5 ppm on each structure. The inset table separates the 42 *N*-glycans into five categories according to their monosaccharide compositions, including high mannose, sialylated, fucosylated, sialylated and fucosylated, and other structures. Divided by the total abundance of all 42 *N*-glycans, the percentage of each category was calculated and is presented by pie chart in Figure 4B. Among these, the sialylated *N*-glycans were the most abundant structures extracted from egg yolks with 65.5% of total abundance. This is followed by 20.7% of other structures and 8% of sialylated + fucosylated glycans. Four high mannose *N*-glycans were detected in egg yolks, including HexNAc_2_Hex_3_ (2300), HexNAc_2_Hex_5_ (2500), HexNAc_2_Hex_6_ (2600), and HexNAc_2_Hex_8_ (2800), which contributed 3.8% to the total abundance. The fucosylated *N*-glycans accounted for 2.1%, making this the rarest type of *N*-glycans in egg yolks.

## 3. Materials and Methods

### 3.1. Materials and Reagents

Fetal bovine fetuin and human serum were purchased from Sigma-Aldrich (St. Louis, MO, USA). Peptide-N-Glycosidase F (PNGase F) was acquired from New England Biolabs (Ipswich, MA, USA). Pierce BCA Protein Assay Kit was purchased from Themo Scientific (Rockford, IL, USA). MS-grade trypsin/Lys-C mix was acquired from Promega (Madison, WI, USA). Sodium hydroxide (NaOH) beads, dithiothreitol (DTT), iodoacetamide (IAA), ammonium bicarbonate (ABC), ammonium-borane complex, and iodomethane were obtained from Sigma-Aldrich (St. Louis, MO, USA). HPLC-grade methanol, acetonitrile (ACN), dimethyl sulfoxide (DMSO), acetone, and HPLC water were purchased from Fisher Scientific (Fair Lawn, NJ, USA). LC-MS grade formic acid (FA) was acquired from TCI America (Portland, OR, USA). Ethanol was purchased from PHARMCO-AAPER (Brookfield, CT, USA). TopTip C-18 cartridges were acquired from Glygen Corp (Columbia, MD, USA). Household bleach with 8.25% of Sodium hypochlorite (NaClO) was obtained from Clorox (Oakland, CA, USA).

It should be noted that the concentration of NaClO in the bleach will decrease gradually during storage, therefore newly produced bleach with sealed cap is always recommended for the experiment. For an open bottle with a long storage time, especially longer than six months, the actual concentration of NaClO should be determined. A commonly used method to calculate the percentage of NaClO in bleach is to react with iodide and produce iodine, followed by adding sodium thiosulfate (Na_2_S_2_O_3_).

### 3.2. Glycomics Sample Preparation

The workflow for glycomics and proteomics sample preparation is shown in Figure S1. First, an aliquot of 60 μg of bovine fetuin and 18 μL of human serum were obtained and dissolved using 300 μL of 50 mM ABC solution. Then, both fetuin and serum samples were separated into six replicates; each contained 10 μg of fetuin and 3 μL of human serum in 50 μL of 50 mM ABC solution. As previously reported [46,47,48], the denaturation of all fetuin and serum samples were performed at 90 °C for 15 min. After cooling down to room temperature, the proteins were reduced and alkylated by adding DTT and IAA, respectively, followed by tryptic digestion at 37 °C overnight. After this, the release of *N*-glycans was achieved by adding 100 units of PNGase F to each sample and incubated at 37 °C for 18 h.

The six replicates of both samples were then dried using a spin vacuum and divided into two triplicates. The first triplicate was subjected to acetone precipitation to separate peptides or proteins from free glycans. The dried samples from the previous step were resuspended in 20 μL of water, then 100 μL of cold acetone (−20 °C) was added. Following the 18 h storage at −20 °C to precipitate glycans, the samples were centrifuged at 21,100× *g* for 10 min. Two separate layers were observed within each sample, where the top layer was an acetone layer containing hydrophobic peptides/proteins, and the bottom layer was a water layer containing hydrophilic glycans. Therefore, the bottom layer was collected for future glycomics analyses. The other triplicate was filtered through SPE C18 cartridges (TopTip C-18 from Glygen Corp, cartridge capacity: 10–200 µL, sorbent binding capacity: 1000 µg). Again, according to the different hydrophobicities, the peptides/proteins were more readily captured by the packing material, whereas the glycans flowed through the cartridges. The C18 cartridges were preconditioned by loading 50 µL of buffer A (40% water, 60% ACN, and 0.1% FA, *v*/*v*) for three times, followed by adding buffer B (100% water and 0.1% FA) for three times. Each sample was reconstituted in 50 µL of buffer B and loaded into C18 cartridges, followed by centrifuge at 0.5 kg for 2 min. The sample loading was repeated three times to ensure a thorough purification of glycans. Finally, the cartridges were washed again using 50 µL of buffer B, and centrifuged at 1.0 kg for 1 min. The flow-through was then collected and dried.

The glycans collected from acetone precipitation and C18 cartridge filtration were reduced and permethylated following the previously reported protocol [49,50,51,52,53]. Briefly, an aliquot of 10 µL of freshly prepared ammonium-borane complex solution (1 µg/µL) was added to each sample and incubated at 60 °C for 1 h to reduce the reducing ends of glycans. To wash off the excess reducing reagent, an aliquot of 1000 µL methanol was added and dried in the spin vacuum for four times. After reduction, glycans were subjected to solid-phase permethylation. The dried glycans were reconstituted in 30 µL of DMSO plus 1.2 µL of water. An aliquot of 20 µL iodomethane was then added. Next, NaOH beads stored in DMSO were transferred into micro spin columns with addition of another 100 µL DMSO to rinse the column. After spin-down at 1800 rpm for 2 min, the samples were loaded into the spin columns followed by incubation at room temperature for 25 min. Then, another aliquot of 20 µL iodomethane was added, followed by another 15 min incubation. After this, the samples were eluted using a centrifuge at 1800 rpm and dried in a spin vacuum dryer. A resuspension solution consisting of 20% ACN, 80% water, and 0.1% FA (*v*/*v*) was utilized to resuspend the samples prior to LC-MS/MS analysis.

### 3.3. Proteomics Sample Preparation

To assess the protein removal performance of acetone precipitation, bovine fetuin and human serum were employed as examples of standard and complex proteins, respectively. Human serum was first subjected to BCA protein assay. Following the vendor’s procedures, the protein concentration was confirmed. Then, as aforementioned, protein samples were dissolved in 50 mM of ABC buffer, then denatured at 90 °C for 15 min, followed by reduction and alkylation. The pH value of each sample was confirmed to be pH = 8 before adding 1 µg of trypsin. After overnight incubation at 37 °C, 0.2 µL of FA was added to quench the digestion process. The tryptic-digested samples were dried, then treated with acetone precipitation and loaded onto C18 cartridges. Again, the bottom layer (water layer) after acetone precipitation and the flow-through were collected for LC-MS/MS proteomics analysis.

### 3.4. N-Glycan Extractions from Egg Yolks

The application of acetone precipitation on large scale samples was demonstrated with *N*-glycan purification from egg yolks. The releasing method of *N*-glycans was modified from Cumming et al. [26]. Three egg yolks (58 g) were separated from egg white and mixed with 400 mL of water. Then, a 67 mL aliquot of 8.25% NaClO solution was added to the mixture and stirred for 15 min. To stop the reaction process, 0.5 mL of Octanol and 5 mL of FA was added and stirred for another 5 min. The supernatant was collected and dried after centrifuge at 9500× *g* for 30 min. To remove the excess salt from the sample, the dried supernatant was resuspended in 120 mL of water and transferred into dialysis tubing, MWCO: 500–1000 D (Spectrum, New Brunswick, NJ, USA). After dialysis against running water for 4 h, the dialysate volume increased to 500 mL. Then, the pH was adjusted to 9.0 by adding 50% *w*/*w* NaOH solution. Another 6 mL of 8.25% NaClO solution was slowly added into the sample solution over 10 min while stirring. Next, 2 mL of FA was added to stop the reaction and the sample was dried overnight in a spin vacuum. The dried residues were resuspended in 120 mL of water and dialyzed for the second time to remove the remaining salts, then dried again. After the oxidative release of glycans, the samples were subjected to acetone precipitation as aforementioned to purify the glycans, followed by reduction and permethylation as described above.

On the other hand, using the traditional enzymatic method to release glycans from the same amount of proteins, and followed by purification using SPE cartridges, can be approximately 170 times more expensive than the proposed method (Table S2). Therefore, oxidative release coupled with acetone precipitation is more practical and economical when it comes to large scale production of glycans.

### 3.5. LC-MS/MS Conditions

The glycomics and proteomics samples were analyzed by an UltiMate 3000 Nano-LC system (Dionex, Sunnyvale, CA) interfaced with an LTQ Orbitrap Velos or Q Exactive HF Orbitrap mass spectrometer (Thermo Scientific, San Jose, CA). The mobile phase A consisted of 98% HPLC water and 2% ACN, containing 0.1% FA (*v*/*v*). The mobile phase B was 100% ACN with 0.1% FA (*v*/*v*). For glycomics, the glycans were separated using a reversed-phase Acclaim PepMap C18 column (150 mm × 75 μm i.d.) under 55 °C. The flow rate was 0.35 μL/min. The total elution time of the multistep gradient was 60 min, starting with 20% mobile phase B over the first 10 min, then increased to 42% in one minute, gradually elevated to 55% B over the next 37 min, elevated to 90% in one minute and stayed for 5 min, then dropped to 20% B in one minute, and stayed for 6 min to rebalance the column. For proteomics, the same column and flow rate were applied. However, the temperature was set at 29.5 °C. The elution gradient was 120 min, starting at 5% of mobile phase B for the first 10 min, then gradually increased to 20% in 55 min, 20% to 30% in the next 25 min, 30% to 50% in 20 min, then elevated to 80% in 1 min and stayed for 4 min, then decreased to 5% in 1 min and balanced for 4 min.

All analytes were detected by mass spectrometer in positive ion-mode. The glycomics and proteomics analyses of bovine fetuin and human serum were performed on LTQ Orbitrap Velos. There were two scan events, including the full MS scan and MS/MS scan. For full MS, the resolving power was set to 100,000. The mass ranges for *N*-glycomics and proteomics were set at 700–2000 *m/z* and 400–2000 *m/z*, respectively. The mass accuracy was 10 ppm. Then, the eight ions with highest intensities from the full MS scan were picked for CID MS/MS scans. The normalized collision energy (NCE) was 35% and the activation time was 10 ms. The glycans extracted from egg yolks were detected by Q Exactive HF, with the full MS resolving power of 60,000. The mass range was set at 200–1800 *m/z*. Then, the top 20 most abundant precursor ions from the full MS were selected and fragmented using HCD with an NCE of 23% and an activation time of 10 ms.

## 4. Summary

In this study, we compared two sample purification methods for glycomics analysis using acetone precipitation and C18 cartridges. Initially, no significant differences were found between the two methods based on the total *N*-glycan and protein abundance results derived from bovine fetuin. However, since bovine fetuin is a single glycoprotein standard, it is not representative for other types of *N*-glycans or proteins. Therefore, we performed the same comparative experiments on human serum, which is a typical complex biological sample. The glycomics results indicated that acetone precipitation has better performance than C18 cartridges in terms of *N*-glycan abundance, especially for the complex sialylated structures. Although the proteomics results suggest that acetone precipitation was able remove 97.7% of the proteins from tryptic digested human serum, C18 cartridges were more effective with more than 99.9% of proteins removed. According to the TICs of the acetone and C18 treated samples, this was due to the stronger affinity between the hydrophobic analytes and the packing materials in C18 cartridges. The higher hydrophilicity of sialic acid relative to other monosaccharides, making it easier to be precipitated by organic solvent, which could explain the significantly higher abundance of multiple sialylated *N*-glycans using acetone precipitation. On the opposite, the lower hydrophilicity of smaller and neutral glycans can predominantly end up in the acetone layer and cause sample loss. Lastly, we combined acetone precipitation with oxidative release using NaClO, and successfully extracted *N*-glycans from egg yolks. This sample preparation method does not include the usage of enzymes and is more suitable for preparing a large quantity of samples, which can potentially be employed for industrial productions of glycans from natural products.

## Data Availability

The raw data is available on GlycoPost with accession number: GPST000305.

## Supplementary Materials

The following supporting information can be downloaded at: https://www.mdpi.com/article/10.3390/metabo12121285/s1, Table S1: Identified *N*-glycan structures extracted from egg yolks, Table S2: Cost of sample preparation, Figure S1: Workflow of glycomics and proteomics sample preparation, Figure S2: Identification processes of *N*-glycans, Figure S3: TICs of proteomics analyses of 1 μg of bovine fetuin, Figure S4: Comparisons of TICs of tryptic digested bovine fetuin, Figure S5: Total abundances of proteome detected in human serum samples, Figure S6: Comparisons of TICs of tryptic digested human serum containing 10 μg of proteins.
