# Supplementary material for: An Efficient and Economical N-Glycome Sample Preparation Using Acetone Precipitation
Source: Metabolites. 2022 Dec 17;12(12):1285. doi: 10.3390/metabo12121285 (PMC9786591; doi:10.3390/metabo12121285)
Supplement: Supplementary file 1 [file metabolites-12-01285-s001.zip › metabolites-2053192-supplementary.pdf]

Supplementary information

Table of contents

Figure S1. Workflow of glycomics and proteomics sample preparation.

Figure S2. Identification processes of *N*-glycans. (A) EIC of core-fucosylated *N*-glycans with the composition of $HexNAc_4Hex_5Fuc_1$, analyzed using a 150 mm PepMap C18 column. The inset provides the full MS spectrum of this structure. (B) MS/MS spectrum of $HexNAc_4Hex_5Fuc_1$ with fragment ions labeled next to their corresponding peaks. (C) EIC and full MS of sialylated *N*-glycans with the composition of $HexNAc_4Hex_5NeuAc_2$. (D) MS/MS spectrum of $HexNAc_4Hex_5NeuAc_2$. Symbols: , N-acetylglucosamine (GlcNAc); , Galactose (Gal); , Fucose (Fuc); , Mannose (Man); , Glucose (Glc); , N-acetylneuraminic acid (NeuAc/Sialic Acid).

Figure S3. TICs of proteomics analyses of 1 µg of bovine fetuin. (A) TIC of tryptic digested bovine fetuin without acetone of C18 cartridge treatment. (B) TIC of tryptic digested bovine fetuin followed by acetone precipitation. (C) TIC of tryptic digested bovine fetuin followed by filtration using C18 cartridge.

Figure S4. Comparisons of TICs of tryptic digested bovine fetuin after (A) acetone precipitation and (B) C18 cartridge filtration. (C) Bar graph showing average spectra count from three replicates of each sample preparation method. The error bars stand for standard deviations.

Figure S5. TIC comparisons between (A) Tryptic digested human serum; tryptic digested human serum, then treated by acetone precipitation; tryptic digested human serum, then filtered through C18 cartridge. (B) Total abundances of proteome detected in human serum samples prepared by three different methods.

Figure S6. Comparisons of TICs of tryptic digested human serum containing 10 µg of proteins, followed by (A) Acetone precipitation, and (B) C18 cartridge filtration.

Table S1. Identified *N*-glycan structures extracted from egg yolks. The absolute abundance, and mass accuracy of each identified structure are provided. (n=3)

Table S2. Cost of sample preparation (Release glycans from one egg yolk with approx. 2.5g proteins).

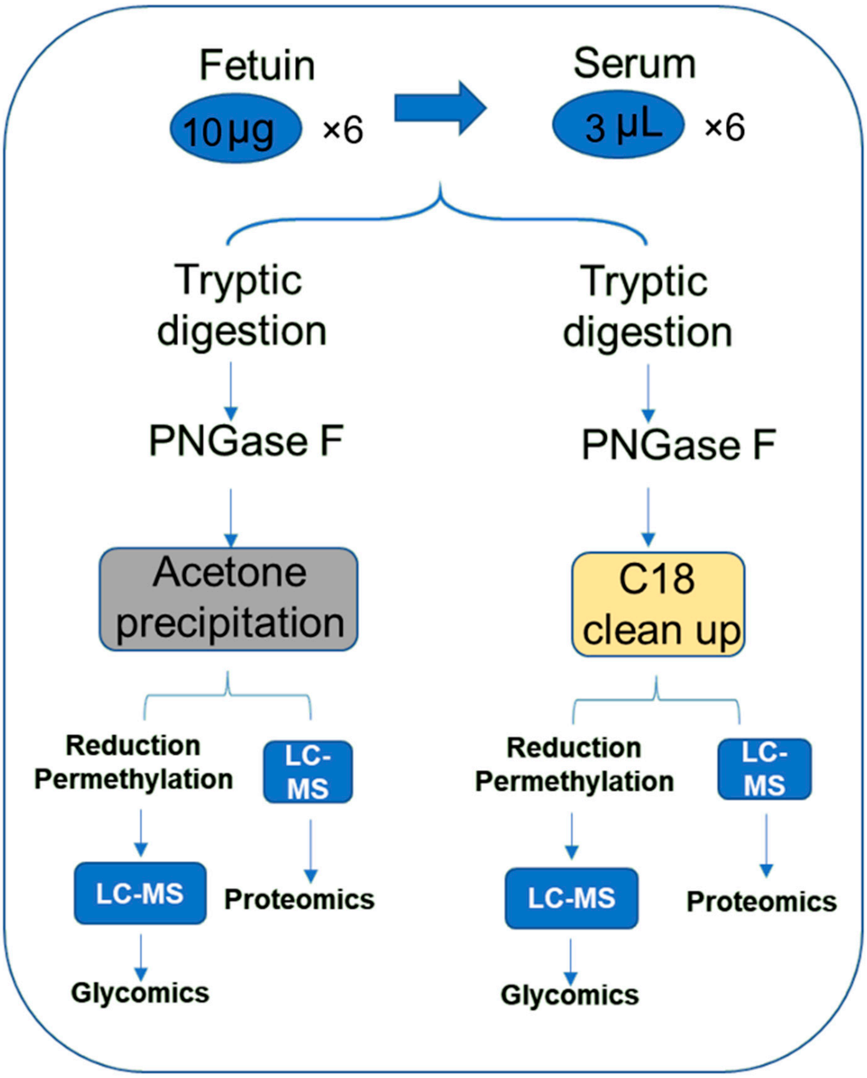

Figure S1. Workflow of glycomics and proteomics sample preparation**.**

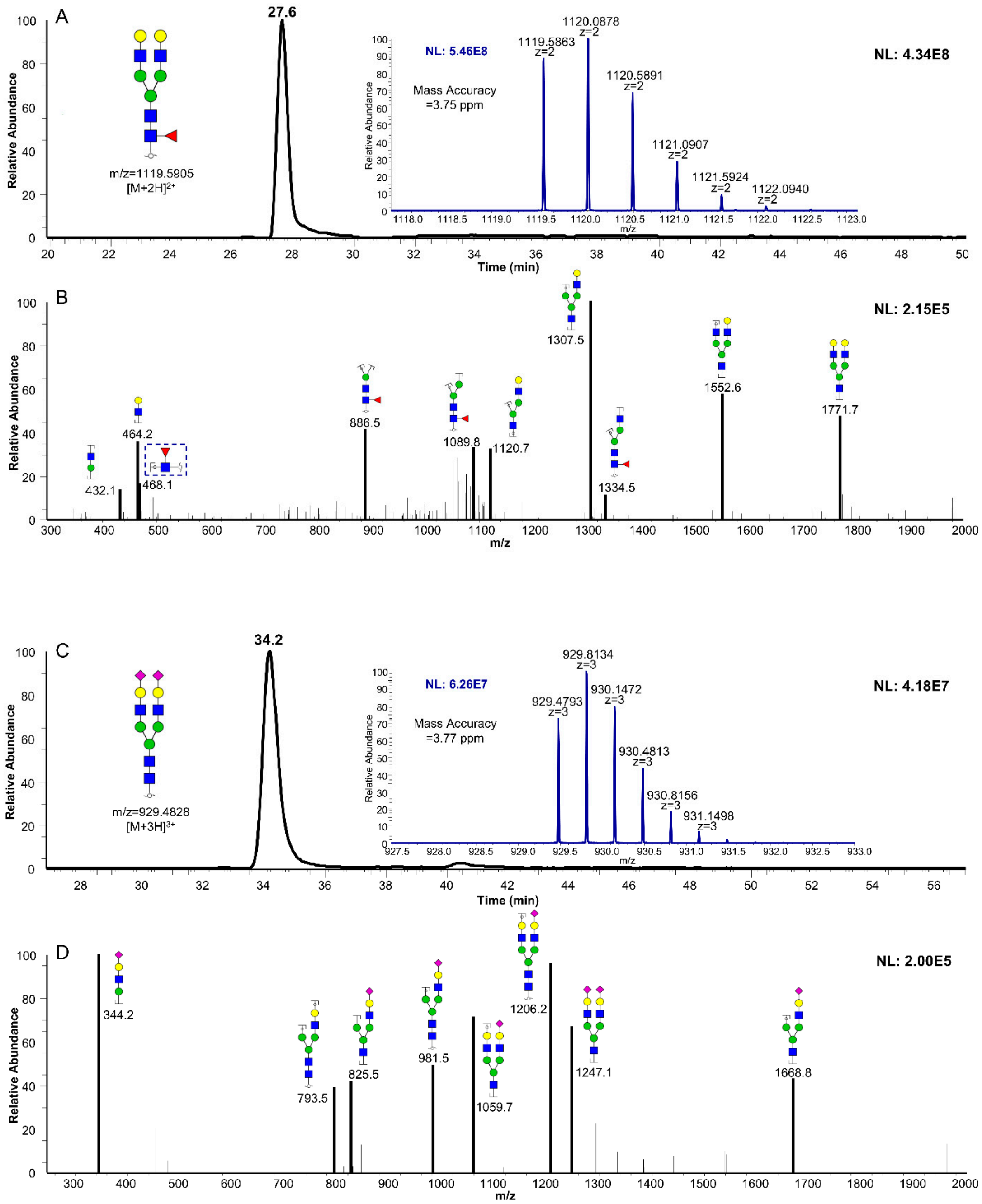

Figure S2. Identification processes of *N*-glycans. (A) EIC of core-fucosylated *N*-glycans with the composition of $HexNAc_4Hex_5Fuc_1$, analyzed using a 150 mm PepMap C18 column. The inset provides the full MS spectrum of this structure. (B) MS/MS spectrum of $HexNAc_4Hex_5Fuc_1$ with fragment ions labeled next to their corresponding peaks. (C) EIC and full MS of sialylated *N*-glycans with the composition of $HexNAc_4Hex_5NeuAc_2$. (D) MS/MS spectrum of $HexNAc_4Hex_5NeuAc_2$. Symbols: ■, N-acetylglucosamine (GlcNAc); ○, Galactose (Gal); ▼, Fucose (Fuc); ●, Mannose (Man); ●, Glucose (Glc); ◆, N-acetylneuraminic acid (NeuAc/Sialic Acid).

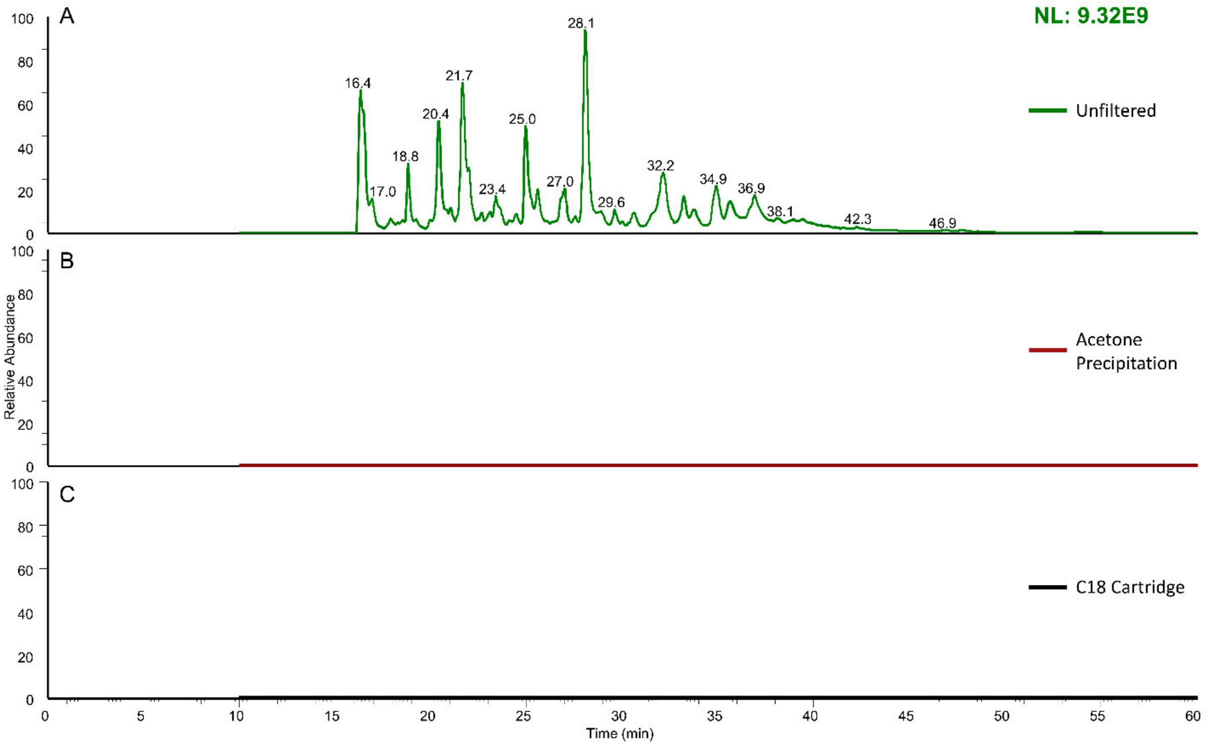

Figure S3. TICs of proteomics analyses of 1 μg of bovine fetuin. (A) TIC of tryptic digested bovine fetuin without acetone of C18 cartridge treatment. (B) TIC of tryptic digested bovine fetuin followed by acetone precipitation. (C) TIC of tryptic digested bovine fetuin followed by filtration using C18 cartridge.

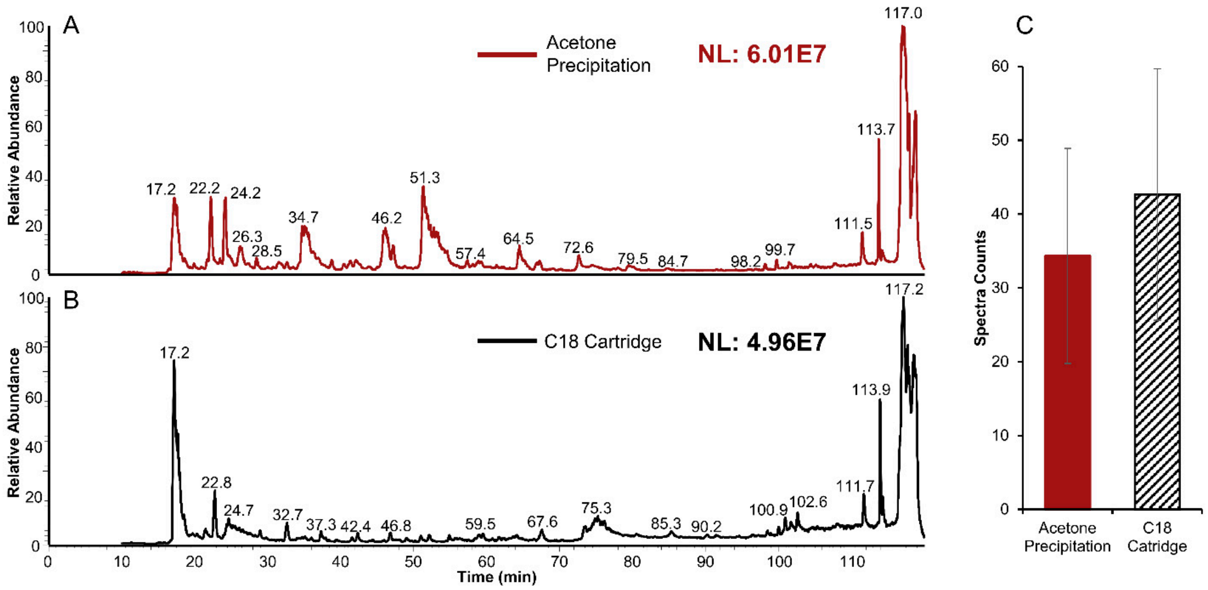

Figure S4. Comparisons of TICs of tryptic digested bovine fetuin after (A) acetone precipitation and (B) C18 cartridge filtration. (C) Bar graph showing average spectra count from three replicates of each sample preparation method. The error bars stand for standard deviations.

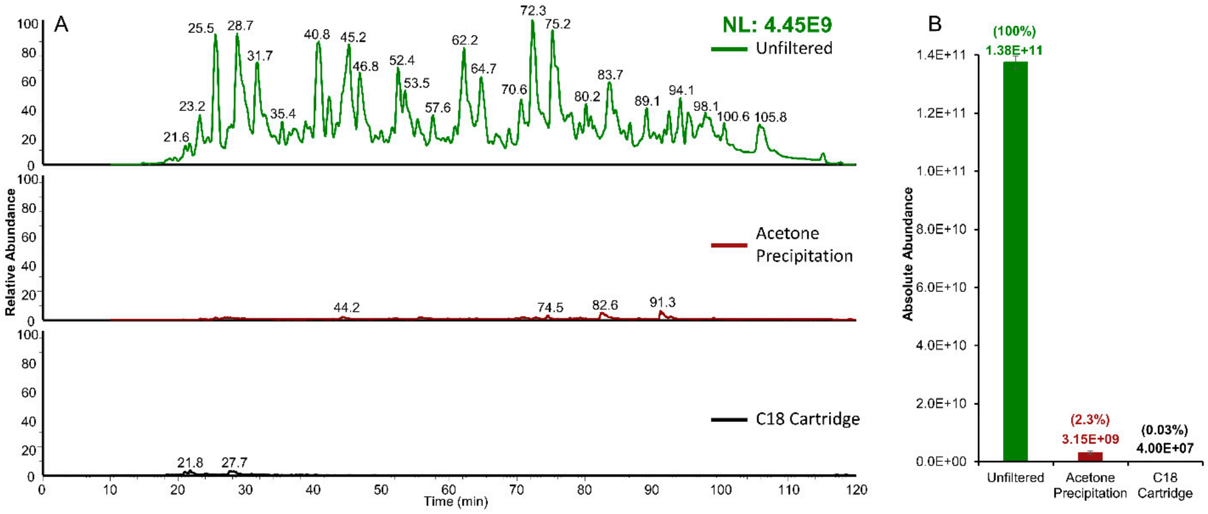

Figure S5. TIC comparisons between (A) Tryptic digested human serum; tryptic digested human serum, then treated by acetone precipitation; tryptic digested human serum, then filtered through C18 cartridge. (B) Total abundances of proteome detected in human serum samples prepared by three different methods.

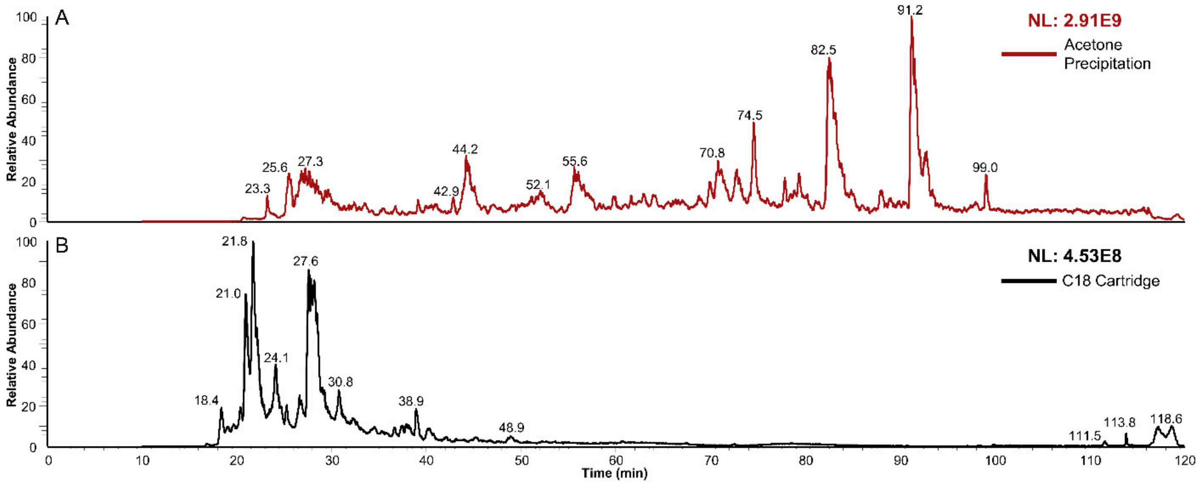

Figure S6. Comparisons of TICs of tryptic digested human serum containing 10 µg of proteins, followed by (A) Acetone precipitation, and (B) C18 cartridge filtration.

Table S1. Cost of sample preparation (release glycans from one egg yolk with approx. 2.5g proteins)

Glycan	Theoretical m/z	Observed m/z	Mass Accuracy (ppm)	Abundance_01	Abundance_02	Abundance_03
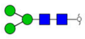	1165.6331	1165.6352	1.80	1.22E+06	1.21E+06	1.40E+06
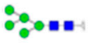	787.4203	787.4217	1.78	2.29E+08	1.93E+08	1.93E+08
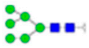	889.4702	889.4720	2.02	2.21E+08	1.79E+08	1.73E+08
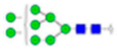	1093.5700	1093.5730	2.74	1.76E+06	2.16E+06	3.45E+06
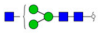	705.8836	705.8842	0.85	1.71E+07	1.32E+07	1.60E+07
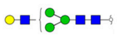	807.9335	807.9348	1.61	1.74E+07	1.95E+07	1.85E+07
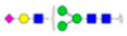	988.5204	988.5219	1.52	8.66E+08	7.10E+08	9.90E+08
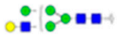	909.9835	909.9843	0.88	1.38E+08	1.29E+08	1.24E+08
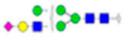	1090.5703	1090.5713	0.92	2.37E+08	1.96E+08	1.86E+08
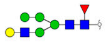	997.0281	997.0323	4.21	7.85E+07	6.88E+07	6.59E+07
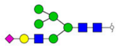	1192.6202	1192.6222	1.68	2.23E+07	1.87E+07	1.83E+07
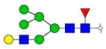	1099.0780	1099.0825	4.09	7.65E+06	5.73E+06	5.85E+06
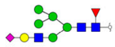	1279.6648	1279.6709	4.77	5.37E+08	4.90E+08	5.26E+08
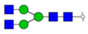	828.4468	828.4488	2.41	2.89E+08	2.74E+08	2.96E+08
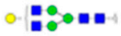	930.4967	930.4979	1.29	1.10E+08	1.07E+08	9.35E+07
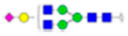	1111.0835	1111.0865	2.70	5.26E+08	4.60E+08	4.81E+08

Glycan	Theoretical m/z	Observed m/z	Mass Accuracy (ppm)	Abundance_01	Abundance_02	Abundance_03
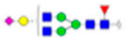	1198.1281	1198.1293	1.00	3.25E+07	3.35E+07	2.93E+07
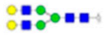	1032.5466	1032.5475	0.87	2.00E+08	1.80E+08	1.76E+08
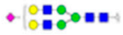	1213.1335	1213.1357	1.81	1.62E+08	1.24E+08	1.36E+08
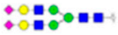	929.4828	929.4848	2.15	4.92E+08	5.03E+08	6.72E+08
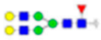	1119.5912	1119.5959	4.20	2.03E+07	8.45E+07	8.60E+07
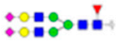	987.5126	987.5145	1.92	8.06E+07	6.96E+07	6.56E+07
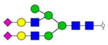	997.5161	997.5170	0.90	1.36E+08	1.53E+08	1.55E+08
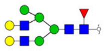	1221.6411	1221.6451	3.27	2.23E+07	1.55E+07	1.70E+07
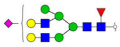	935.1546	935.1569	2.46	1.26E+08	8.86E+07	1.52E+08
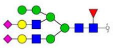	1123.5791	1123.5815	2.14	1.42E+07	1.13E+07	1.09E+07
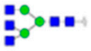	951.0100	951.0115	1.58	7.51E+08	7.41E+08	7.07E+08
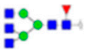	1038.0545	1038.0560	1.45	3.75E+07	4.13E+07	4.07E+07
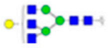	1053.0599	1053.0623	2.28	1.51E+08	1.52E+08	1.49E+08
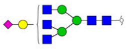	1233.6467	1233.6488	1.70	4.20E+06	3.65E+06	4.00E+06
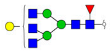	1140.1045	1140.1062	1.49	2.91E+06	2.56E+06	2.81E+06
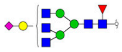	1320.6913	1320.6937	1.82	4.50E+07	5.29E+07	5.00E+07

Glycan	Theoretical m/z	Observed m/z	Mass Accuracy (ppm)	Abundance_01	Abundance_02	Abundance_03
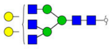	1155.1098	1155.1105	0.61	8.95E+06	5.86E+06	8.53E+06
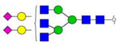	1011.1916	1011.1932	1.58	1.15E+07	9.96E+06	9.12E+06
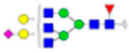	948.8301	948.8326	2.63	1.43E+07	1.18E+07	1.28E+07
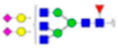	1069.2213	1069.2231	1.68	9.56E+06	7.96E+06	8.84E+06
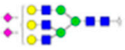	1079.2249	1079.2286	3.43	4.87E+06	4.98E+06	5.81E+06
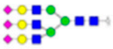	1199.6161	1199.6180	1.58	5.94E+07	4.91E+07	4.66E+07
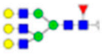	896.4721	896.4730	1.00	1.19E+07	1.20E+07	9.44E+06
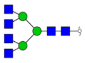	1073.5731	1073.5758	2.51	4.82E+08	4.22E+08	4.80E+08
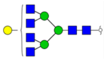	1175.6230	1175.6266	3.06	6.33E+07	5.94E+07	4.40E+07
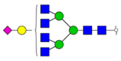	1356.2098	1356.2113	1.11	2.33E+07	1.63E+07	2.06E+07

Table S2. Cost of sample preparation (release glycans from one egg yolk with approx. 2.5g proteins)

Step	Cost of Traditional Method	Cost of the Proposed Method
**Tryptic Digestion**	$ 89,500 (50 mg trypsin)	Not Needed
**Glycan Release**	$ 81,200 (7,500,000 units PNGase F)	$ 20 (One bottle of bleach)
**Glycan Purification**	$ 1,000 (500 C18 cartridges)	$ 350 (4L HPLC Acetone)
**Dialysis**	Not Needed	$ 600 (Dialysis Tube)
**Total Cost**	**> $ 170,000**	**< $ 1,000**
